# Estrogen Decreases Cytoskeletal Organization by Forming an ERα/SHP2/c-Src Complex in Osteoclasts to Protect against Ovariectomy-Induced Bone Loss in Mice

**DOI:** 10.3390/antiox10040619

**Published:** 2021-04-17

**Authors:** Hyun-Jung Park, Malihatosadat Gholam-Zadeh, Sun-Young Yoon, Jae-Hee Suh, Hye-Seon Choi

**Affiliations:** 1 Department of Biological Sciences (BK21 Program), University of Ulsan, Ulsan 680-749, Korea; oli_jjung@naver.com (H.-J.P.); ma_gholamzadeh@yahoo.com (M.G.-Z.); tjsdud9981@naver.com (S.-Y.Y.); 2 Department of Pathology, Ulsan University Hospital, Ulsan 682-714, Korea; drjhsuh@gmail.com

**Keywords:** estrogen (E_2_), ERα, osteoclast, actin ring formation, non-genomic signal

## Abstract

Loss of ovarian function is closely related to estrogen (E_2_) deficiency, which is responsible for increased osteoclast (OC) differentiation and activity. We aimed to investigate the action mechanism of E_2_ to decrease bone resorption in OCs to protect from ovariectomy (OVX)-induced bone loss in mice. In vivo, tartrate-resistant acid phosphatase (TRAP) staining in femur and serum carboxy-terminal collagen crosslinks-1 (CTX-1) were analyzed upon E_2_ injection after OVX in mice. In vitro, OCs were analyzed by TRAP staining, actin ring formation, carboxymethylation, determination of reactive oxygen species (ROS) level, and immunoprecipitation coupled with Western blot. In vivo and in vitro, E_2_ decreased OC size more dramatically than OC number and Methyl-piperidino-pyrazole hydrate dihydrochloride (MPPD), an estrogen receptor alpha (ERα) antagonist, augmented the OC size. ERα was found in plasma membranes and E_2_/ERα signaling affected receptor activator of nuclear factor κB ligand (RANKL)-induced actin ring formation by rapidly decreasing a proto-oncogene tyrosine-protein kinase, cellular sarcoma (c-Src) (Y416) phosphorylation in OCs. E_2_ exposure decreased physical interactions between NADPH oxidase 1 (NOX1) and the oxidized form of c-Src homology 2 (SH2)-containing protein tyrosine phosphatase 2 (SHP2), leading to higher levels of reduced SHP2. ERα formed a complex with the reduced form of SHP2 and c-Src to decrease c-Src activation upon E_2_ exposure, which blocked a signal for actin ring formation by decreased Vav guanine nucleotide exchange factor 3 (Vav3) (p–Y) and Ras-related C3 botulinum toxin substrate 1 (Rac1) (GTP) activation in OCs. E_2_/ERα signals consistently inhibited bone resorption in vitro. In conclusion, our study suggests that E_2_-binding to ERα forms a complex with SHP2/c-Src to attenuate c-Src activation that was induced upon RANKL stimulation in a non-genomic manner, resulting in an impaired actin ring formation and reducing bone resorption.

## 1. Introduction

Within the skeleton, constant remodeling and repairing of old bones is required to ensure structural integrity. Excessive bone resorption leads to decreased bone mass, disrupted architecture, or inappropriate bone formation responses during remodeling [1]. Bone resorbing cells, osteoclasts (OCs) require two essential factors; macrophage-colony stimulating factor (M-CSF) and RANKL. M-CSF stimulates mainly OCs survival and proliferation as well as activation through cross-talking with RANKL [2]. A family member of tumor necrosis factor receptor, RANK expresses on OC precursor cells as a transmembrane signaling receptor for RANKL to result in expression of OC-specific genes, activation of bone resorption, and OC survival [3]. The degree of bone resorption reflects the number and matrix-degrading activity of OCs [4]. The number of OCs is regulated by OC differentiation as well as OC survival. While the functional activity of mature OCs in bone resorption is mainly governed by the cytoskeletal organization of an actin ring structure that surrounds the resorption area, isolating it from the extracellular space and concentrating bone-degrading molecules. OCs express a receptor for vitronectin, integrin alpha V beta 3, which plays a critical role in cytoskeletal organization. A deficiency of α_V_β_3_ integrin has been reported to disrupt cytoskeletal organization, leading to increased bone mass caused by impaired bone resorption by OCs [5]. The absence of c-Src has also been demonstrated to result in impaired cytoskeletal organization, similar to the phenotype of α_V_β_3_ integrin-deficiency [6]. More recently, the signaling complex of c-Src/Vav3 was found to be activated upon integrin occupancy [7] to activate Rac1 [8]. RANKL has been reported to directly promote bone resorption by OC via inducing the association of c-Src with receptor activator of nuclear factor κB (RANK) in an α_V_β_3_ integrin-dependent manner. Cross-linking of RANKL and RANK induces an interaction with α_V_β_3_ to induce an integrin-associated canonical pathway for actin ring formation via an axis of c-Src/Vav3/Rac1 activation [9].

Postmenopausal osteoporosis is a systemic skeletal disease involving low bone mass and deteriorated microarchitecture because of a loss of ovarian function. The decline of ovarian function has been associated with estrogen (E_2_) deficiency, which increases the formation and activity of osteoclasts (OCs). Elevated osteoclastogenesis, caused by a drop in estrogen levels, is the most common characteristic of postmenopausal osteoporosis [10,11]. Although E_2_ has mainly been considered to affect the attenuation of bone resorption to prevent bone loss [4,12], the detailed mechanisms involved remain to be elucidated.

In our study, we investigated an action mechanism of E_2_ signaling that is associated with decreased bone resorption in OCs.

## 2. Materials and Methods

### 2.1. Ethics Statement

All mice were handled following guidelines of the Institutional Animal Care and Use Committee (IACUC) of the Immunomodulation Research Center (IRC), University of Ulsan. All animal procedures were approved by the IACUC of IRC. The approval ID for this study is #HSC-19–010 (20190801).

### 2.2. Reagents and Antibodies

Recombinant mouse macrophage-colony stimulating factor (M-CSF) and receptor activator of nuclear factor κB ligand (RANKL) were obtained from R and D Systems, Inc. (Minneapolis, MN, USA). Estradiol, 2′,2′,2′-tribromoethanol, *N*-acetyl-l-cysteine (NAC), and Hoechst 33258 were obtained from Sigma Chemicals (St. Louis, MO, USA). MPPD was from Tocris (Bristol, UK). Minimum essential medium α (α-MEM) without phenol red and MicroAmp fast reaction tubes (8 tubes/strip) were from Life Technologies (Carlsbad, CA, USA). We obtained rhodamine–phalloidin from Molecular Probes (Carlsbad, CA, USA) and *N*-(biotinoyl)-*N*′-(iodoacetyl) ethylenediamine (BIAM) from Invitrogen (Carlsbad, CA, USA). Abs against phospho-tyrosine (4G10) was purchased from Upstate USA Inc. (Charlottesville, VA, USA), and Vav guanine nucleotide exchange factor 3 (Vav3) was from Abcam (Cambridge, MA, USA). c-Src-Y416 was obtained from Cell Signaling Technology (Denver, MA, USA); c-Src homology 2 (SH2) containing protein tyrosine phosphatase 2 (SHP2), estrogen receptor α (ERα), and caveolin-1 were from Santa Cruz Biotechnology (Santa Cruz, CA, USA); c-Src was sourced from ECM Biosciences (Versailles, KY, USA); NADPH oxidase 1 (NOX1) was from Novus (Centennial, CO, USA); and β-actin was from Sigma-Aldrich (St. Louis, MO, USA). In accordance with the manufacturer’s protocol, active Ras-related C3 botulinum toxin substrate (Rac) was measured using the Rac1 activation kit from Thermo Scientific (Rockford, IL, USA). The RatLaps kit (carboxy-terminal collagen crosslinks-1; CTX-1) EIA was from Immunodiagnostic Systems Inc. (Fountain Hills, AZ, USA).

### 2.3. Animals, Culture of OCs, and OC Formation

Ten-week-old female an inbred strain, C57 black 6 J (C57BL/6 J) mice were subjected to a sham operation (*n* = 10) or ovariectomy (OVX) (*n* = 12) under anesthesia using 2,2,2-tribromoethanol (250 mg/kg). E_2_ (0.1 mg/kg) or vehicle was injected intraperitoneally and daily for 4 weeks starting 2 days after surgery. Blood was collected retro-orbitally under anesthesia before sacrifice, and tissues were harvested immediately. In vivo markers of bone resorption were measured according to the manufacturer’s directions (Immunodiagnostic Systems Inc., Fountain Hills, AZ, USA) and serum CTX-1 was assessed using a RatLaps enzyme-linked immunosorbent assay (EIA). To determine TRAP-positive OCs in vivo, mouse femora were excised, cleaned with soft tissue, and decalcified in ethylenediaminetetraacetic acid (EDTA). Representative histological sections of the distal femoral metaphysis from mice in each of the four groups were stained for TRAP to identify OCs (original magnification ×400).

The femora and tibiae were removed aseptically and dissected to remove adherent soft tissue. The ends of the bones were cut, and the marrow cavity was flushed with α-MEM from one end using a sterile 21-gauge needle. The bone marrow was further agitated using a Pasteur pipette to obtain a single-cell suspension, which was washed twice and incubated on culture plates with M-CSF (20 ng/mL) for 16 h (h) Non-adherent cells were then harvested, layered on a Ficoll–Hypaque gradient, and cultured for 2 more days, by which time large populations of adherent monocyte/macrophage-like cells had formed on the bottoms of the culture plates, as previously described [13]. The few non-adherent cells were removed by washing the dishes with phosphate-buffered saline (PBS), and the adherent cells (bone marrow-derived macrophages (BMMs)) were harvested and seeded onto culture plates. The adherent cells were analyzed as negative for a T cell coreceptor, cluster of differentiation 3 (CD3) and a member of protein tyrosine phosphatase expressed on B cells, CD45R, and positive for an M-CSF receptor, cluster of differentiation 115 (CD115) [14]. The absence of contaminating stromal cells was confirmed by the lack of cell growth in the absence of M-CSF. Additional medium containing M-CSF and RANKL (40 ng/mL) was added, and the medium was replaced on day 3. For E_2_ treatment in vitro, the BMMs were cultured in α-MEM without phenol red containing 10% charcoal-treated fetal bovine serum (FBS) [15]. After incubation for the indicated times, the cells were fixed in 10% formalin for 10 min and stained for TRAP as described [13]. The numbers of TRAP-positive multinucleated cells (MNCs) (three or more nuclei) were recorded. The area and maximum diameter of the formed OCs were measured, and the fusion index was presented as the average number of nuclei per TRAP-positive MNC [16].

### 2.4. RNA Isolation and Quantitative Polymerase Chain Reaction (qPCR)

Total RNA was isolated using QIAzol reagent. The first-strand cDNA was reverse-transcribed with random primers and Moloney murine leukemia virus (M-MLV) reverse transcriptase as described in Park et al. [17]. qPCR was carried out using SYBR green real-time PCR master mixes and the appropriate primers. Relative gene expression was calculated using the formula 2^−∆∆Ct^ with normalization to ribosomal protein small subunit (RPS) gene that has been known for housekeeping [18]. The primer sequences were used as described [17].

### 2.5. Actin Cytoskeleton

To examine the actin ring within the OCs, mature OCs were cultured for 4 h under the indicated conditions as described in Kim et al. [19]. The slides were treated with 0.1% Triton X-100 in PBS for 5 min and stained with rhodamine–phalloidin for actin and Hoechst33258 for the nuclei. The cytoplasmic distribution of nuclei and F-actin were examined using an Olympus FV1200 confocal microscope (Olympus, Tokyo, Japan).

### 2.6. Western Blot Analysis

Cultured cells were harvested after washing with ice-cold PBS and then lysed in extraction buffer (50 mM Tris-HCl, pH 8.0, 150 mM NaCl, 1 mM EDTA, 0.5% Nonidet *P*-40, 0.01% protease inhibitor mixture). The protein concentration was determined using bicinchoninic acid (BCA) assay. Cell extracts (20 μg) were subjected to sodium dodecyl sulfate–polyacrylamide gel electrophoresis (SDS–PAGE) and transferred onto nitrocellulose membranes. Membranes were blocked for 1 h with skim milk in Tris-buffered saline containing 0.1% Tween-20% and incubated overnight at 4 °C [17] with antibodies (Ab) against c-Src-Y416, c-Src, and β-actin. An active pull-down and detection kit was used to extract and detect active Rac1 (89856Y), as directed by the manufacturer. The lysate (200 μg) was subjected to immunoprecipitation with 1 µg of antibody against Vav3, ERα, c-Src, or SHP2, followed by Western blot analysis using the corresponding Ab as indicated. Membranes were washed, incubated for 1 h with horseradish peroxidase (HRP)-conjugated secondary antibodies, and developed using chemiluminescence substrates. The original images for Western blots have been provided (Supplementary Figures S1–S3).

### 2.7. Detection of Oxidized SHP2 by Carboxymethylation

BMMs were incubated with M-CSF (30 ng/mL) and RANKL (40 ng/mL) for 55 h and further incubated in the presence or absence of E_2_ (5 nM) for 16 h. The medium was removed, and the cells were frozen rapidly in liquid nitrogen. The frozen cells were transferred to 100 µM *N*-(biotinoyl)-*N′*-(iodoacetyl) ethylenediamine (BIAM)-containing lysis buffer (50 mM Tris-HCl, pH 7.5, 150 mM NaCl, 0.5% Triton X-100, 10 µg/mL aprotinin, and 10 µg/mL leupeptin; rendered oxygen-free by bubbling nitrogen gas through the buffer at a low flow rate for 20 min). Sulfhydryl modifying chemical BIAM selectively detects the reduced form of cysteine [20]. After sonication in a bath sonicator for three 1-min periods, the lysate was clarified by centrifugation and subjected to immunoprecipitation with 1 µg of Ab against SHP2. Immunocomplexes labeled with BIAM were detected with HRP-conjugated streptavidin, and the color was developed with an enhanced chemiluminescence kit.

### 2.8. Flow-Cytometric Quantification of Intracellular Reactive Oxygen Species (ROS)

The intracellular ROS was detected using the fluorescent probe, 2′7′-dichlorofluorescein diacetate (H_2_DCFDA) [21]. Incubated cells in the presence or absence of E_2_ (5 nM), MPPD (2 μM) for 16 h, washed thoroughly, stained with H_2_DCFDA at 37 °C for 30 min. Intracellular ROS was measured by fluorescence-activated cell sorting (FACS) Calibur flow cytometer (Becton Dickinson, Franklin Lakes, NJ, USA).

### 2.9. Bone Resorption

OCs were further characterized by assessing their ability to form pits on dentine slices, as described in an earlier report [17]. To this end, mature OCs, which were generated by treating BMMs with M-CSF (30 ng/mL) and RANKL (40 ng/mL), were seeded on dentine slices and further incubated with E_2_ or MPPD for another 3 days. Cells were fixed with formalin and stained for TRAP. Then, the cells were removed by ultrasonication in 1 M NH_4_OH and stained with 1% (*w*/*v*) toluidine blue in 0.5% sodium borate to visualize resorption pits. The resorption pit area was measured with ImageJ software, 1.37v.

### 2.10. Statistical Analysis

Values are expressed as means of triplicate experiments ± standard deviation (SD). Each series of experiments was repeated at least three times. Statistical analysis was performed by Student’s *t*-test when two groups were compared. Two-way analysis of variance (ANOVA) was used when two variables were analyzed. A *p*-value of less than 0.05 was considered statistically significant.

## 3. Results

### 3.1. E_2_ Decreases Number and Size of OCs during Bone Loss in OVX Mice

**Figure 1:**
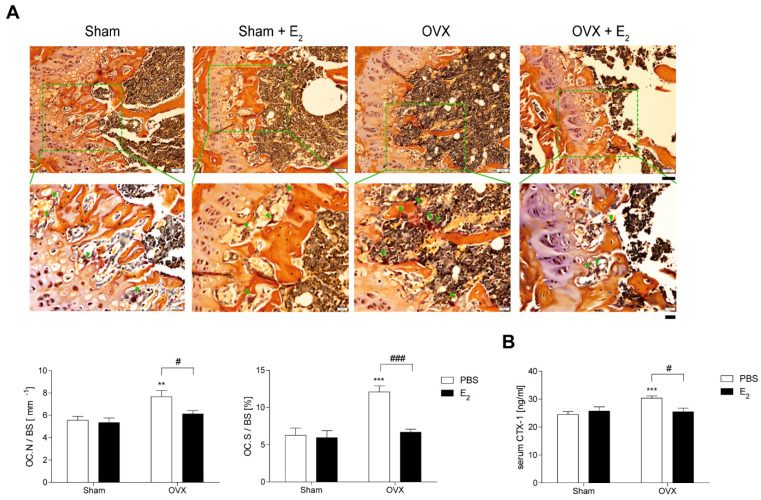
E_2_ decreases number and size of osteoclasts (OCs) during bone loss in ovariectomy (OVX) mice. (**A**) Representative histological sections of the distal femoral metaphysis of mice from each of the 4 groups were stained for tartrate-resistant acid phosphatase (TRAP) to identify OCs (indicated by arrows) to calculate OC surface area divided by total bone surface area (OC.S/BS) and OC number divided by total bone surface (OC.N/BS). Scale bar in the representative photos: 50 μm. Inset shows higher magnification (scale bar: 20 μm). (**B**) Serum carboxy-terminal collagen crosslinks-1 (CTX-1) in vivo bone resorption marker. ** *p* < 0.01; *** *p* < 0.001 compared with sham mice. ^#^ *p* < 0.05; ^###^ *p* < 0.001 compared with PBS-injected mice. Two-way ANOVA, followed by Bonferroni posttests was used to compare the effect of E_2_ (OC.N/BS and serum CTX-1; *p* < 0.05, OC.S/BS; *p* < 0.001), the effect of surgery (serum CTX-1; *p* < 0.05, OC.N/BS; *p* < 0.01, OC.S/BS; *p* < 0.001 ) and interactions (OC.S/BS; *p* < 0.01, serum CTX-1; *p* < 0.05) (**A**,**B**).

To investigate the role of E_2_ in OVX-induced bone loss, we evaluated the effect of E_2_ on OCs from E_2_-injected OVX mice. In vivo, TRAP-staining showed that E_2_ significantly decreased OC surface area divided by total bone surface area (OC.S/BS), which increased after 4 weeks of OVX with a modest decrease in OC number divided by total bone surface (OC.N/BS) (Figure 1A). A similar pattern was observed in vivo with the bone-resorption marker, serum CTX-1 (Figure 1B).

### 3.2. E_2_ Inhibits NUMBER and Size of OCs during Osteoclast Differentiation

**Figure 2:**
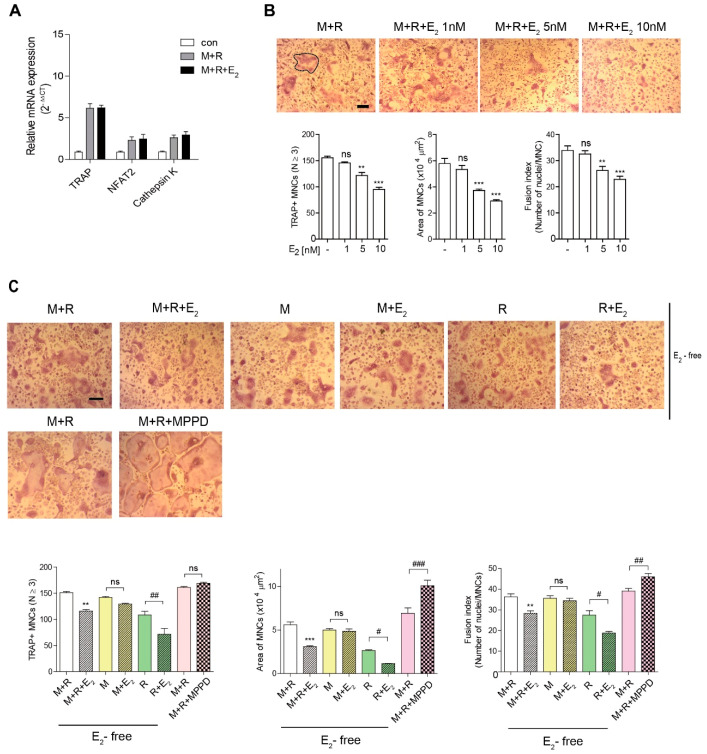
E_2_ inhibits the number and size of OCs during osteoclast differentiation in vitro. (**A**) RNA from bone marrow-derived macrophages (BMMs) cultured with M-CSF (30 ng/mL) and receptor activator of nuclear factor κB ligand (RANKL) (40 ng/mL) in the presence of E_2_ (5 nM) for 48 h was analyzed by qPCR. The expression level before RANKL treatment was set to 1. (**B**,**C**) BMMs were cultured with M-CSF (30 ng/mL) and RANKL (40 ng/mL) for 5 h, and then E_2_ (1 nM, 5 nM, 10 nM) (**B**) or MPPD (2 μM) (**C**) was added for another 16 h upon M-CSF and/or RANKL to determine TRAP-positive multinucleated cells (MNCs). After cells were fixed, more than 70 TRAP-positive MNCs in each culture were randomly selected. The area of formed OCs surrounded by the bold line was measured. The fusion index presented is the average number of nuclei per TRAP-positive MNC formed in the culture. Representative photos are shown. Scale bar in the representative OC photos: 100 μm. ** *p* < 0.01; *** *p* < 0.001 compared with vehicle group. ^#^ *p* < 0.05; ^##^ *p* < 0.01; ^###^ *p* < 0.001 compared with each corresponding control. Similar results were obtained in three independent experiments.

To assess the effect of E_2_ on OC differentiation in vitro, we determined the expression of OC-specific genes on RANKL stimulation after 48 h of exposure to E_2_. As shown in Figure 2A, E_2_ exposure did not change the RANKL-induced OC differentiation-associated genes expression levels, including TRAP, nuclear factors of activated T cells 2 (NFAT2), calcineurin- and calcium-regulated transcription factor, and the lysosomal proteolytic enzyme cathepsin K [2,3]. To confirm that E_2_ acts in the late stages of osteoclastogenesis, we added E_2_ to OC cultures after 55 h exposure of RANKL. E_2_ decreased the number of OCs, the OC area, and fusion index at the late-stage and had profound effects on OC size, compared to number and fusion index (Figure 2B). Next, we assessed which cytokine signal was specific to E_2_ exposure. The effect of E_2_ on the OC area was more dramatic upon RANKL stimulation, whereas there was no significant effect of E_2_ on M-CSF stimulation (Figure 2C). Conversely, blocking the E_2_ signal with the ERα antagonist, MPPD, increased OC area and fusion index without any change in the OC number (Figure 2C). MPPD more efficiently augmented OC area than it did fusion index and number of OCs.

### 3.3. E_2_ Inhibits RANKL-Stimulated Actin Ring Formation

**Figure 3:**
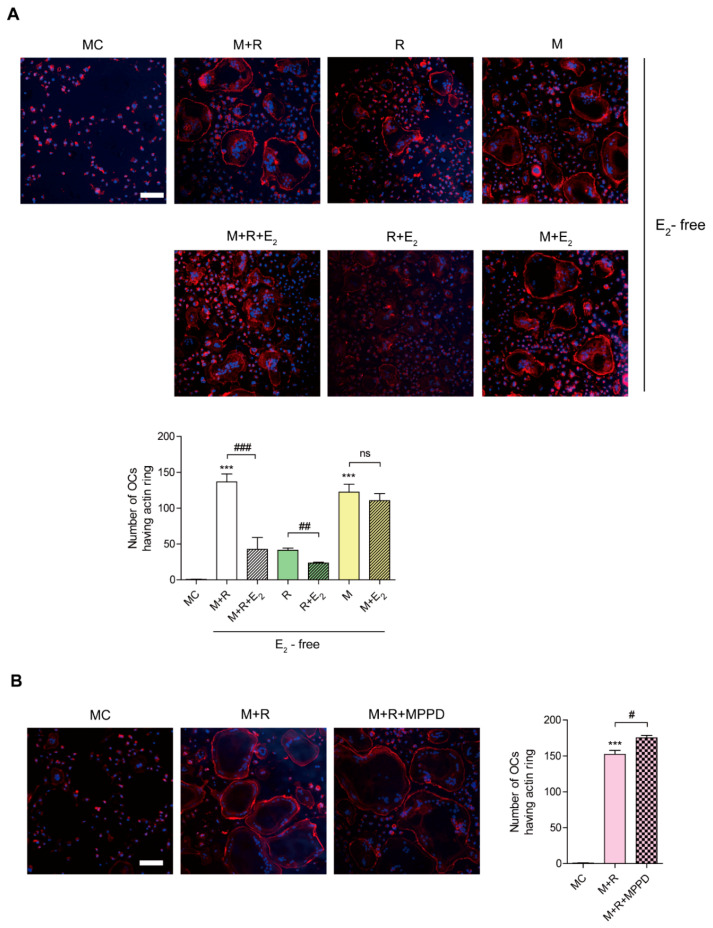
E_2_ inhibits RANKL-stimulated actin ring formation. BMMs were cultured with M-CSF (30 ng/mL) and RANKL (40 ng/mL) for 96 h. (**A**) Cells were incubated with α-MEM/10% FBS (media control, MC) or with E_2_ (5 nM) in the presence of M-CSF (M, 30 ng/mL) and/or RANKL (R, 40 ng/mL) as indicated. (**B**) Cells were incubated with α-MEM/10% FBS (media control, MC) or with MPPD (2 μM) in the presence of M-CSF and RANKL. After a 4 h incubation, the cells were stained with rhodamine–phalloidin and Hoechst to visualize actin ring and nuclei, respectively. (**A**,**B**) show representative images. Scale bar, 100 μm. The number of OCs having actin rings was plotted for the indicated conditions. *** *p* < 0.001 compared with MC. ^#^ *p* < 0.05; ^##^ *p* < 0.01; ^###^ *p* < 0.001 compared with each corresponding control. Similar results were obtained in three independent experiments.

To confirm whether E_2_ affects OC spreading by impairing actin cytoskeletal reorganization, we evaluated whether E_2_ inhibits the actin ring formation essential for bone resorption in OCs. Mature OCs were generated on a plastic well and incubated with M-CSF or RANKL in the presence or absence of E_2_, and the cells were stained with rhodamine–phalloidin to visualize the actin ring. As shown in Figure 3A, the removal of cytokines completely abolished actin ring-containing OCs, whereas the addition of M-CSF and RANKL recovered the number of actin ring-containing cells. However, E_2_ reduced it. The effect of E_2_ on actin-ring formation was more prominent with RANKL stimulation, whereas no significant effect was observed with M-CSF stimulation. In contrast, MPPD treatment increased the number of OCs having actin rings (Figure 3B).

### 3.4. E_2_ Transmits Signaling by Forming an ERα/c-Src/SHP2 Complex, Resulting in Disrupted c-Src Activation in a Non-Genomic Manner

**Figure 4:**
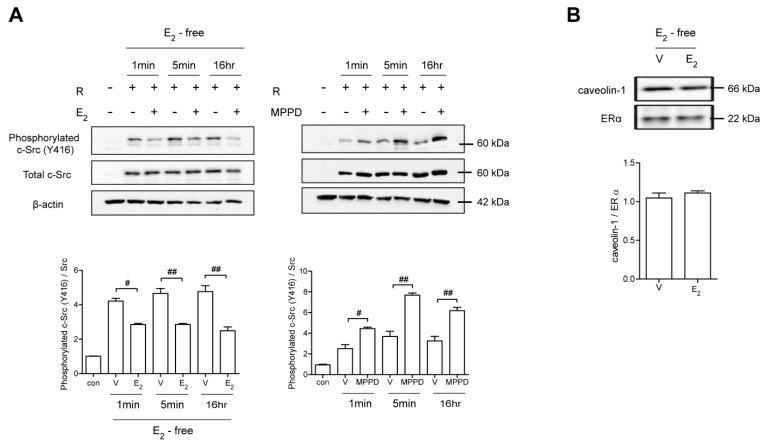
E_2_ transmits signaling via ERα, resulting in disrupted c-Src activation in a non-genomic manner. BMMs stimulated with RANKL (50 ng/mL) in the absence and presence of E_2_ (5 nM) or MPPD (2 μM) for the indicated time. (**A**) The cell lysate was immunoblotted for phosphorylated c-Src (Y416). (**B**) Cell lysates were prepared for co-immunoprecipitation with specific antibodies to ERα and subjected to immunoblotting as indicated. ^#^ *p* < 0.05; ^##^ *p* < 0.01 compared with each corresponding control. Similar results were obtained in three independent experiments.

**Figure 5:**
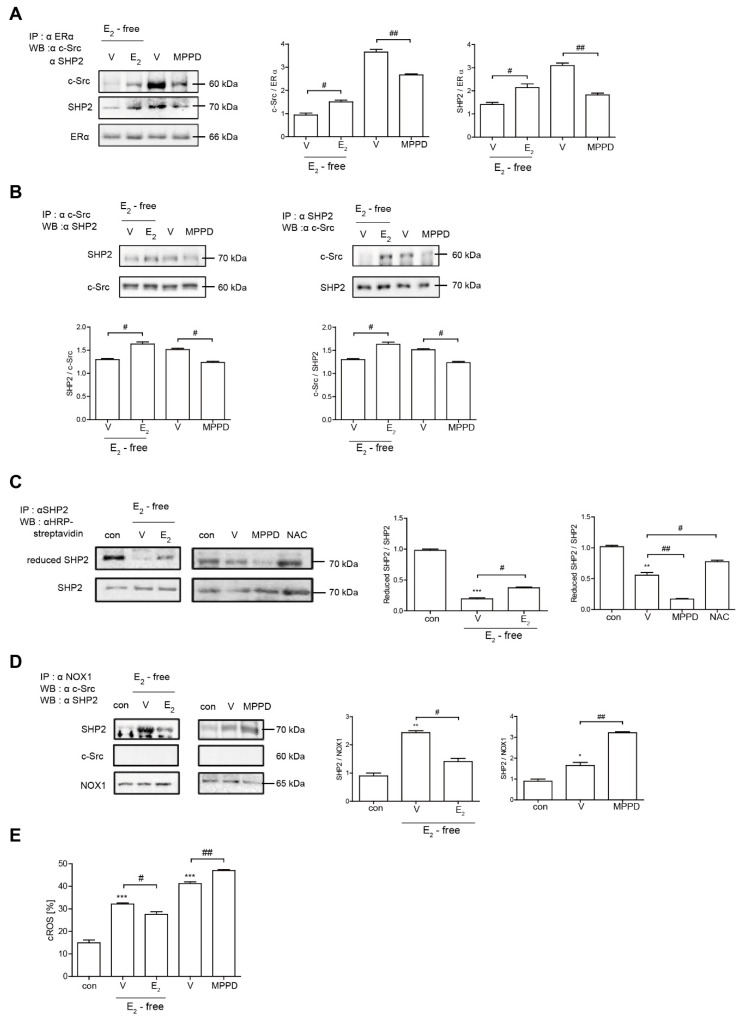
E_2_ transmits signaling by forming an ERα/c-Src/SHP2 complex to decrease c-Src activation. (**A**,**B**,**D**) Cell lysates were prepared for co-immunoprecipitation with specific antibodies to ERα, c-Src, SHP2, or NOX1 and subjected to immunoblotting as indicated. (**C**) After labeling of cell lysate using BIAM, immunoprecipitation (IP) was performed with anti-SHP2, followed by HRP–streptavidin immunoblotting to isolate the reduced form of SHP2. (**E**) BMMs were cultured with M-CSF and RANKL for 55 h, and then E_2_ (5 nM) or MPPD (2 μM) was added for another 16 h upon M-CSF and/or RANKL to determine cytosolic ROS level. * *p* < 0.05; ** *p* < 0.01; *** *p* < 0.001 compared with control. ^#^ *p* < 0.05; ^##^ *p* < 0.01 compared with each corresponding control. Similar results were obtained in three independent experiments.

Because E_2_ more potently blocked RANKL-induced actin ring formation in OCs, we evaluated the effect of E_2_ on RANKL-stimulated signaling pathways that mediate cytoskeletal reorganization. RANKL-induced c-Src activation was evaluated by the phosphorylation of c-Src-Y416. E_2_ has been reported to transmit hormonal signals through genomic or non-genomic mechanisms [22,23,24]; therefore, we determined the times required for E_2_ to decrease c-Src activation. As shown in Figure 4A, E_2_ significantly reduced the amount of phosphorylated c-Src as early as 1 min exposure. Whereas the ERα antagonist, MPPD, increased phosphorylated c-Src (Supplementary Figure S1). Western blot analysis combined with co-immunoprecipitation showed a direct interaction between ERα and caveolin-1, a plasma membrane marker, in the absence or presence of E_2_ in OCs (Figure 4B, Supplementary Figure S1). Next, we determined whether ERα associates with c-Src to generate an E_2_ response upon RANKL stimulation in OCs. As shown in the top panel of Figure 5A, co-immunoprecipitation demonstrated that the direct interaction between ERα and c-Src upon RANKL stimulation was enhanced after E_2_ exposure, whereas it was attenuated upon MPPD treatment. Since tyrosine phosphatase is required to decrease c-Src activation, and SHP2 has been demonstrated to have a physical association with ERα [23], we evaluated whether this is the case when E_2_ was added in RANKL-stimulated OCs. As we expected, there was an increased association between SHP2 and ERα upon E_2_ exposure. The opposite result was observed with MPPD treatment (Figure 5A, Supplementary Figure S2). E_2_ was then shown to enhance the direct interaction between c-Src and SHP2 in the co-immunoprecipitation experiment with SHP2, followed by binding of c-Src, (Figure 5B, left, Supplementary Figure S2). The same phenomenon was observed with immunoprecipitation with c-Src and immunoblotting with SHP2 (Figure 5B, right, Supplementary Figure S2). Next, to investigate how the activity of SHP2 is regulated, we evaluated whether ROS affects the activity of SHP2 via oxidation. We labeled the cell with BIAM, which is a sulfhydryl-modifying reagent that exhibits selective binding with the thiolate form of reduced cysteine (Cys) residues. SHP2 was immunoprecipitated and biotinylated, and reduced fractions of SHP2 were conjugated with HRP–streptavidin. As shown in Figure 5C, SHP2 was oxidized upon RANKL stimulation, as there were decreased levels of the reduced form of SHP2, whereas E_2_ exposure reversed this effect. MPPD increased the oxidization of SHP2, whereas *N*-acetyl cysteine (NAC), a ROS scavenger, decreased it as a positive control (Supplementary Figure S2). Then, to find out the potential molecule that contributes to converting SHP2 by ROS generation, we examined whether SHP2 was modulated by its interaction with NOX1 upon E_2_ exposure. As shown in Figure 5D, RANKL induced a direct interaction between SHP2 and NOX1, whereas E_2_ decreased their association. The opposite was seen with MPPD treatment. However, NOX1 did not interact with c-Src in the presence of E_2_ or MPPD (Supplementary Figure S2). Next, we determined ROS generated from NOX1 upon exposure to E_2_ or MPPD to assess the activity of NOX1. RANKL alone increased ROS, whereas the addition of E_2_ decreased it, and MPPD reversed it (Figure 5E).

### 3.5. E_2_ Inhibits RANKL-Induced Cytoskeletal Reorganization via an Axis of c-Src/Vav3/Rac1, Leading to Decreased Bone Resorption

**Figure 6:**
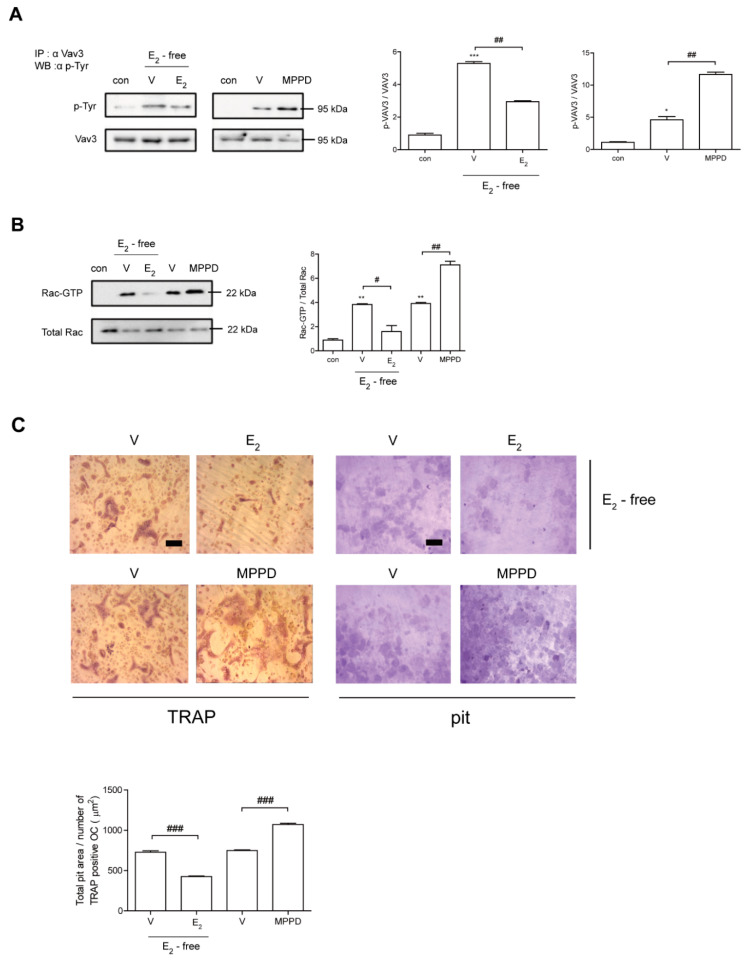
E_2_ inhibits RANKL-induced cytoskeletal reorganization via an axis of c-Src/Vav3/Rac1, leading to decreased bone resorption. (**A**) Vav3 was immunoprecipitated from cell lysate, and the phosphotyrosine (p-Tyr) content of the immunoprecipitate was determined by immunoblot with anti-phosphotyrosine Ab. (**B**) GTP-Rac was isolated by GST pull-down and immunoblotted with Rac-specific Ab. (**C**) Mature OCs were incubated further on whole dentine slices with M-CSF and RANKL in the presence or absence of E_2_ (5 nM) or MPPD (2 μM) for 3 days. After TRAP staining, the cells were removed, and the slices were stained with toluidine blue. Representative photos of TRAP-positive OCs and resorption pits are shown. Scale bar in the representative photos: 100 μm. The total pit area/number of TRAP-positive OCs was calculated. * *p* < 0.05; ** *p* < 0.01; *** *p* < 0.001 compared with control. ^#^ *p* < 0.05; ^##^ *p* < 0.01; ^###^ *p* < 0.001 compared with each corresponding control. Similar results were obtained in three independent experiments.

Next, we assessed whether decreased c-Src activation is transmitted to block activations of Vav3 and Rac1 to affect actin ring formation. Vav3 has been reported to be an OC-specific guanidine nucleotide exchange factor that targets Rac1 [25]. Consistent with its effect on c-Src, E_2_ attenuated the tyrosine phosphorylation of Vav3 induced by RANKL (Figure 6A, Supplementary Figure S3). Rac1 activation was assessed using a glutathione-S-transferase (GST) pull-down assay. While Rac1 activation was enhanced after 5 min exposure to RANKL, it was reduced by the addition of E_2_ compared with RANKL alone (Figure 6B, Supplementary Figure S3). Consistent with its morphological and functional phenotypes, E_2_ suppressed the major cytoskeleton-organizing signals by decreasing c-Src/Vav3/Rac1 signaling in OCs.

To determine whether E_2_ affects OC activity, we assessed the effect of E_2_ on bone resorption using dentine slices. As shown in Figure 6C, mature OCs generated with M-CSF and RANKL were mounted on dentine slices with/without E_2_ in the presence of cytokines. The addition of E_2_ resulted in significantly reduced OC total pit area/OC number compared with cells stimulated with cytokines only, whereas MPPD increased it (Figure 6C).

## 4. Discussion

We have demonstrated the mechanisms by which E_2_ affects the OC to recover OVX-induced bone loss. E_2_ did not change the expression of OC-specific genes, such as TRAP, NFAT2, and cathepsin K, suggesting that the early stages of OC differentiation were not affected by E_2_. However, E_2_ decreased the cell area more prominently than it did the number and fusion index of OCs, while the opposite pattern was observed with the ERα antagonist, MPPD, implying the possibility that E_2_/ERα signaling may reduce OC spreading via disrupted cytoskeletal reorganization. As we expected, E_2_ impaired actin ring formation. The signaling through E_2_/ERα was affected upon RANKL stimulation but not upon M-CSF stimulation, suggesting that impaired OC spreading by E_2_ signals could be RANKL-dependent. Our data demonstrated that E_2_ decreased RANKL-induced signaling for cytoskeletal reorganization through blocking the activation of c-Src/Vav3/Rac1, an effect that also has been reported for RANKL stimulation [9]. After 1 min of E_2_ exposure, the level of phosphorylated c-Src-Y416 was diminished, suggesting that the signaling was transmitted rapidly. In addition, the co-immunoprecipitation assay showed there was an interaction between ERα and caveolin-1, a plasma membrane marker, indicating ERα is located on plasma membranes. Those findings suggested that the inhibitory effect of E_2_ during RANKL-induced c-Src activation occurred in a non-genomic way. Although ERs belong to the nuclear receptor protein family, which regulates the expression of target genes by binding DNA at specific response elements, many studies have demonstrated these receptors to have secondary signaling roles transmitted in a non-genomic way [22,23,24]. In agreement with our results, E_2_ exhibits rapid cellular responses in a non-nuclear manner, acting through receptors found in cell membranes as well as in the cytoplasm [22]. The rapid signaling of E_2_, via the association between ERα and c-Src, also has been demonstrated in endothelial cells [24].

The protective effects of E_2_ in bone loss have been reported to occur through OCs [26,27]. OC-specific ERα-knockout mice exhibited a similar phenotype to that of osteoporotic women with low trabecular bone density and failed to show further bone loss upon loss of ovarian function [27]. In the absence of ERα, mature OCs are resistant to the apoptotic effects of E_2_, implying that the main role of E_2_ in OCs is to enhance apoptosis and thereby increase bone density [27]. In agreement with this, the effect of E_2_ on bone resorption has been suggested to involve inducing OCs to directly carry out apoptosis [28,29]. However, our results demonstrated that E_2_ more potently decreased OC activity by impairing cytoskeletal organization than it did OC number under the assayed conditions. Our results corroborate those of several studies that found E_2_ affects the cytoskeleton in OCs [30,31]. The absence of Siglec-15 exhibited resistance to E_2_ deficiency-induced bone loss with OCs that failed to spread onto the bone surface, indicating that E_2_ is associated with cytoskeleton organization via Siglec-15 in OCs [30]. Genistein, a phytoestrogen, disrupted actin ring formation by elevating cytosolic Ca^2+^ concentrations, resulting in attenuated bone resorption in rat OCs [31].

We demonstrated the detailed molecular mechanism of how E_2_ disrupted cytoskeletal reorganization in OCs. E_2_ enhanced the physical association between c-Src and the reduced (active) form of SHP2. E_2_/ERα formed a complex with c-Src and SHP2, resulting in the dephosphorylation of Y416 of c-Src. Thus, the direct coupling of ERα to both c-Src and SHP2 acted to dampen the signaling event triggered by RANKL. The positive role of SHP2 in mediating E_2_ signaling by forming an SHP2/ERα complex has been reported in the modulation of body weight and energy balance in conjunction with leptin [23]. In addition, the SHP2 effect was enhanced in Shp^D61A^ mutants that have increased catalytic activity [23], suggesting the important role of SHP2 activity in E_2_/ERα signaling. We hypothesized that ROS modulates SHP2 activity via its interaction with NOX1 demonstrated to be induced upon RANKL stimulation [32]. The assays of the immunoprecipitation, carboxymethylation, and ROS level showed that RANKL stimulation increased a direct interaction between NOX1 and SHP2 inactive ROS-induced oxidation, whereas E_2_ exposure reversed it. A similar pattern was found with NAC, suggesting the effect of E_2_ was mediated by decreased ROS levels. Taken together, the results showed that E_2_/ERα transmits the signal to form a complex with active SHP2 and c-Src due to decreased interaction between NOX1 and inactive SHP2, finally leading to attenuating c-Src activation upon RANKL stimulation.

Although E_2_ exhibits strong protective effects against postmenopausal osteoporosis in clinical studies, its therapeutic application has been limited due to its side effects [33]. Our results suggest that SHP2 or NOX1 acts as a downstream molecule to exhibit the E_2_ effect in OCs. Currently, the number of SHP2 inhibitors are under clinical trials for tumor-targeted therapies [34] and NAC as a ROS scavenger has been reported to improve traumatic brain injury in human trials [35], suggesting an implication for their therapeutic application as an alternative to E_2_ for bone loss in human.

## 5. Conclusions

**Figure 7:**
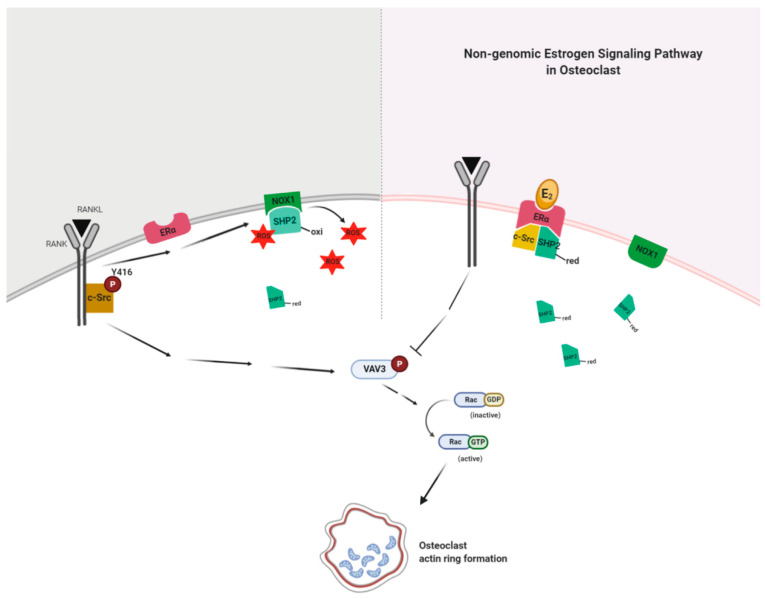
E_2_ signaling plays a critical role in an impaired actin ring formation. RANKL activates c-Src and transmits a signal to generate reactive oxygen species (ROS) via NADPH oxidase 1 (NOX1), resulting in the association of NOX1 and the oxidized form (inactive) of c-Src homology 2 (SH2)-containing protein tyrosine phosphatase 2 (SHP2) to decrease the availability of the reduced (active) form of SHP2. E_2_ binding to ERα forms a complex with active SHP2 and c-Src to attenuate RANKL-stimulated c-Src activation due to increased availability of the reduced form of SHP2 through the decreased interaction between NOX1 and the oxidized form of SHP2 in a non-genomic way. Dephosphorylation of c-Src is followed by the blockade of Vav3 and Rac1 activation by RANKL stimulation. This results in impaired actin ring formation in OCs and, therefore, reduced bone resorption.

Our present findings suggest that E_2_ binding to ERα formed a complex with active SHP2 and c-Src to attenuate RANKL-stimulated c-Src activation due to decreased interaction between NOX1 and inactive SHP2 in a non-genomic way. Dephosphorylation of c-Src was followed by the blockade of Vav3 and Rac1 activation by RANKL stimulation (Figure 7). This resulted in impaired actin ring formation in OCs and, therefore, reduced bone resorption. Our results demonstrate the novel action mechanism of E_2_ in OCs to impair cytoskeletal reorganization in a non-genomic way, suggesting that SHP2 or NOX1 could be a potential therapeutic target for osteoporosis upon loss of ovarian function.

## Data Availability

All original images and data are contained within the article.

## Supplementary Materials

The following are available online at https://www.mdpi.com/article/10.3390/antiox10040619/s1, Figure S1: Raw data of western blot for Figure 4, Figure S2: Raw data of western blot for Figure 5, and Figure S3: Raw data of western blot for Figure 6.
