# Supplementary figures and images for: Estrogen Decreases Cytoskeletal Organization by Forming an ERα/SHP2/c-Src Complex in Osteoclasts to Protect against Ovariectomy-Induced Bone Loss in Mice
Source: Antioxidants (Basel). 2021 Apr 17;10(4):619. doi: 10.3390/antiox10040619 (PMC8073670; doi:10.3390/antiox10040619)

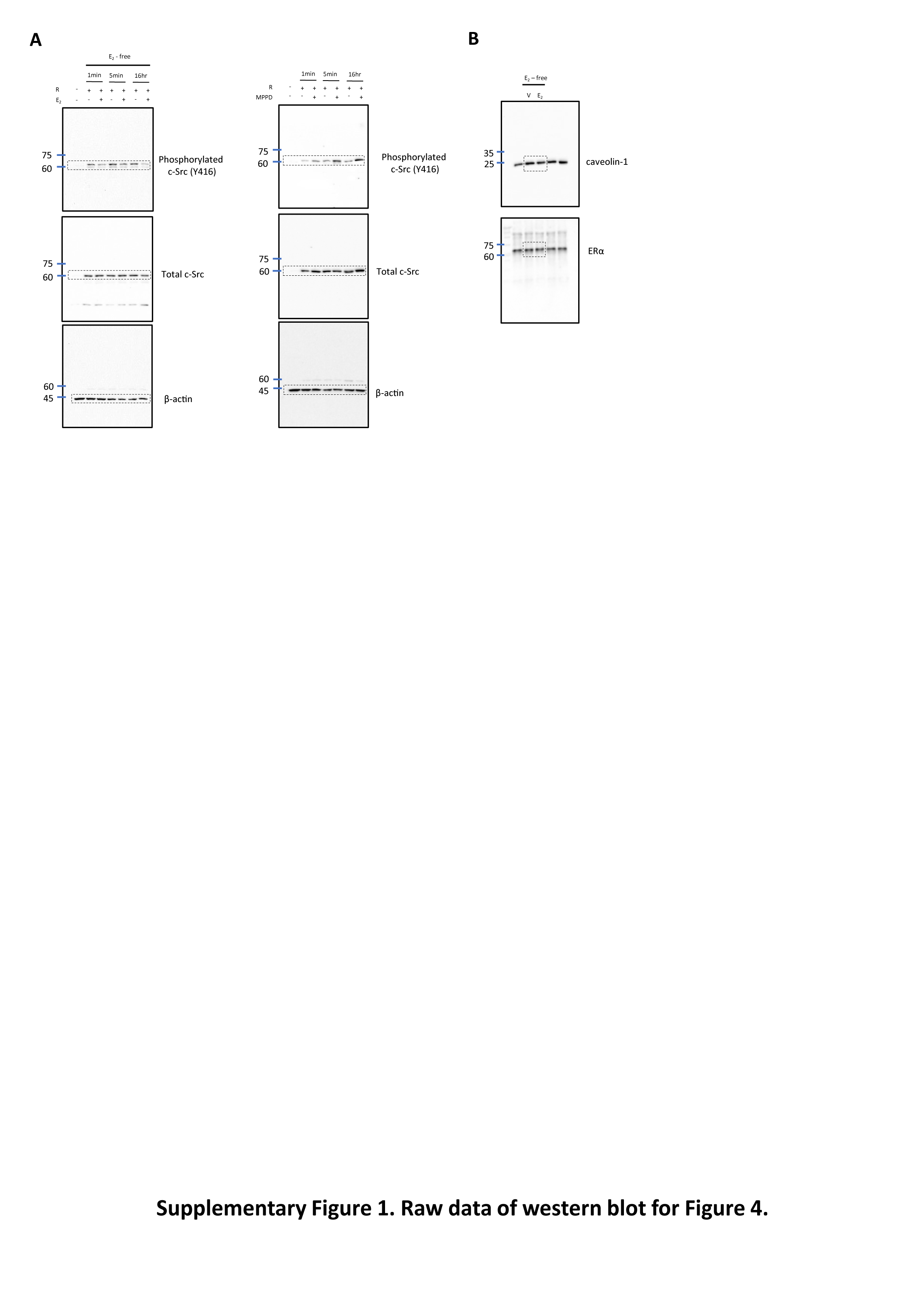

Supplement: Supplementary file 1 [file antioxidants-10-00619-s001.zip › Supplementary Figure S1.tif]

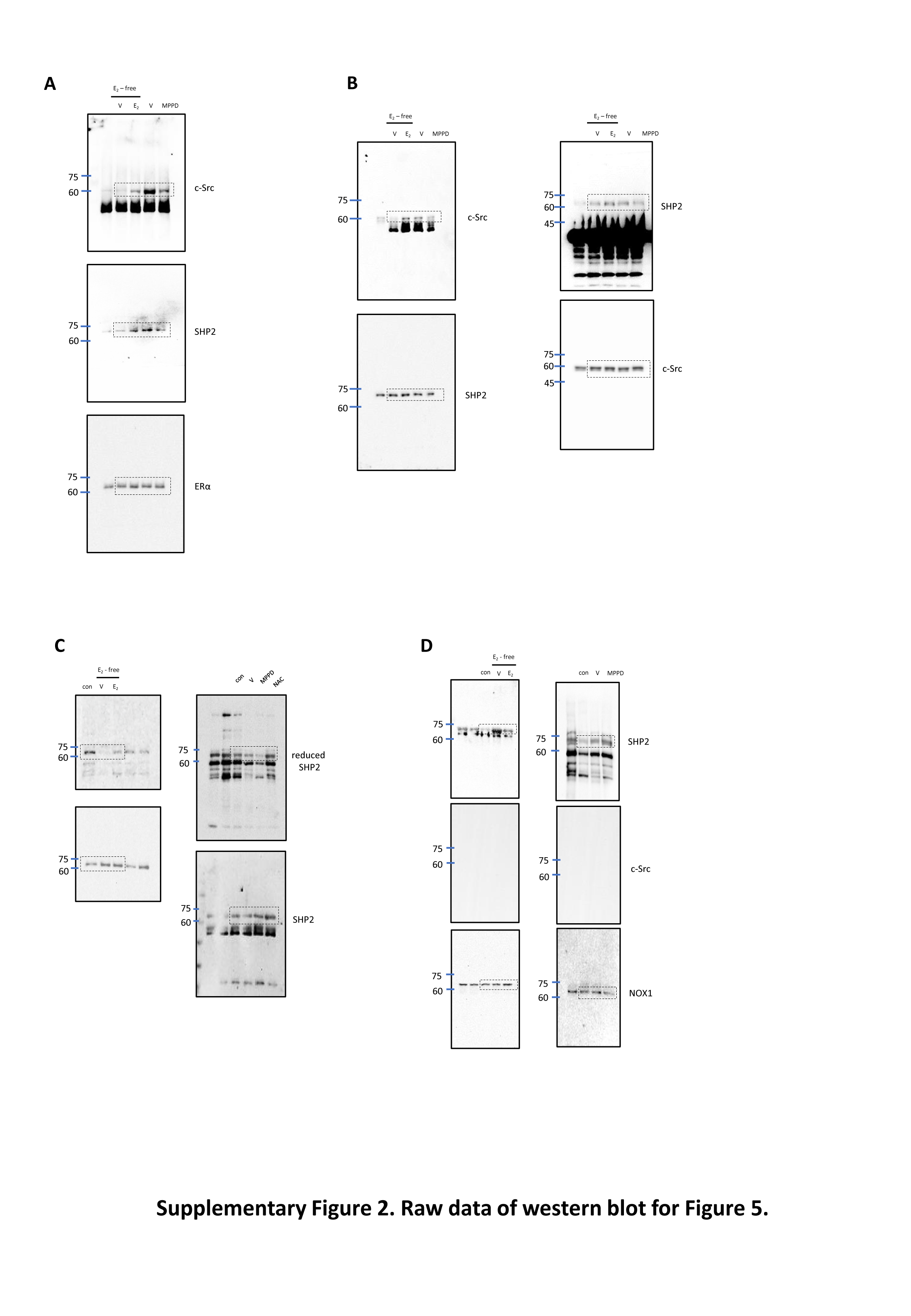

Supplement: Supplementary file 1 [file antioxidants-10-00619-s001.zip › Supplementary Figure S2.tif]

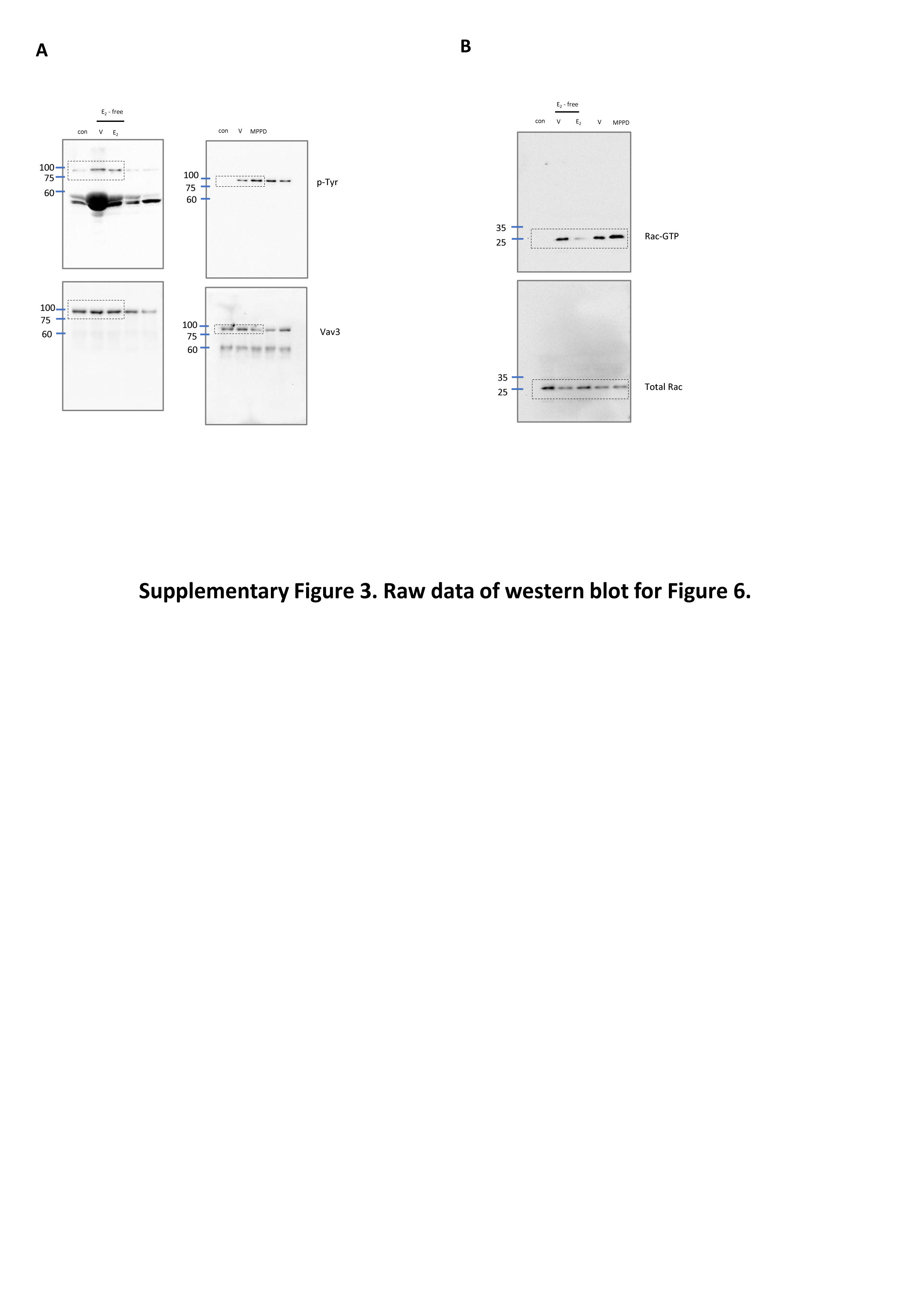

Supplement: Supplementary file 1 [file antioxidants-10-00619-s001.zip › Supplementary Figure S3.tif]
